# Use of a Text Message Program to Raise Type 2 Diabetes Risk Awareness and Promote Health Behavior Change (Part II): Assessment of Participants' Perceptions on Efficacy

**DOI:** 10.2196/jmir.2929

**Published:** 2013-12-19

**Authors:** Lorraine R Buis, Lindsey Hirzel, Scott A Turske, Terrisca R Des Jardins, Hossein Yarandi, Patricia Bondurant

**Affiliations:** ^1^University of MichiganDepartment of Family MedicineAnn Arbor, MIUnited States; ^2^Wayne State UniversityCollege of NursingDetroit, MIUnited States; ^3^Southeast Michigan Beacon CommunityDetroit, MIUnited States; ^4^Greater Cincinnati Beacon CollaborativeCincinnati, OHUnited States

**Keywords:** diabetes mellitus, type 2, mobile health, cellular phone, text messaging, risk reduction behavior, program evaluation

## Abstract

**Background:**

Although there is great enthusiasm in both the public and private sector for the further development and use of large-scale consumer-facing public health applications for mobile platforms, little is known about user experience and satisfaction with this type of approach. As a part of the Beacon Community Cooperative Agreement Program, txt4health, a public-facing, mobile phone-based health information service targeting type 2 diabetes, was launched in 3 Beacon Communities: the Southeast Michigan Beacon Community in Detroit, MI, the Greater Cincinnati Beacon Community in Cincinnati, OH, and the Crescent City Beacon Community in New Orleans, LA. This program was marketed via large public health campaigns and drew many users within the respective communities.

**Objective:**

The purpose of this investigation was to use the RE-AIM framework to document txt4health efficacy by focusing on perceptions of satisfaction, usage, and behavior change among individuals who used txt4health in pilot studies in Southeast Michigan and Greater Cincinnati.

**Methods:**

We conducted a multimodal user survey with txt4health users recruited via text message through the program to understand participant perceptions of program use and satisfaction, as well as self-reported perceptions of behavior change as a result of using txt4health.

**Results:**

Txt4health users reported very high levels of program satisfaction, with 67.1% (108/161) reporting satisfaction scores of ≥8 on a 10-point scale, with 10 equivalent to most satisfied (mean 8.2, SD 1.6). All survey participants agreed/strongly agreed that the messages included in txt4health were clear and easy to understand (100.0%, 160/160), and most found txt4health made them knowledgeable about their risk for type 2 diabetes (88.1%, 140/159) and made them conscious of their diet and physical activity (88.8%, 142/160). Most participants reported that txt4health helped them to make behavior changes related to diet; after having completed txt4health, most agreed/strongly agreed that they are more likely to replace sugary drinks, such as juice or soda, with water (78.0%, 124/159), have a piece of fresh fruit instead of dessert (74.2%, 118/159), substitute a small salad for chips or fries when dining out (76.1%, 121/159), buy healthier foods when grocery shopping (79.7%, 126/158), and eat more grilled, baked, or broiled foods instead of fried (75.5%, 120/159).

**Conclusions:**

Results from this study suggest that participants in txt4health, a large-scale, public health–focused text message program targeting type 2 diabetes, have positive perceptions of the program and that participation has led to positive behavior change.

## Introduction

In an era of health care reform occurring at a time when cell phones and mobile devices are near ubiquitous within the United States population [1], it is not surprising to see the rapid expansion of mobile health (mHealth) for the delivery of health information and services. Although there is great enthusiasm in both the public and private sector for the further development and use of consumer-facing health-related applications for mobile platforms, the momentum of mHealth is quickly outpacing the evidence base documenting the efficacy of these endeavors, as well as the traditional research designs used in the creation of the evidence base [2-4]. The efficacy of text message-based interventions to promote behavior change within the context of chronic illness has been previously documented within small-scale contexts [5-7], but most of the available evidence base is focused on underpowered small-scale pilot studies with few participants [4-6]. This fact leads many to believe that, similar to other innovations within the health care industry, moving beyond the pilot phase to operating at scale is an issue plaguing the mHealth field today.

Because of the affordances of a large-scale text message campaign, such as wide reach, low participant costs, and a small time investment on the part of participants, these types of programs appear to be perfectly suited to broad-based public health campaigns. Txt4baby is an example of this type of public health approach [8], which has garnered significant attention in the popular press and many people are attempting to replicate its successes. Txt4health, a text message program that offers participants a type 2 diabetes risk assessment followed by 14 weeks of tailored messaging focused on behavior changes for physical activity and diet, is one such program that was piloted in the Detroit, MI, Cincinnati, OH, and New Orleans, LA metropolitan areas through funding from the Beacon Community Cooperative Agreement Program. Although text message programs are well documented at the pilot stage, large-scale applications in the United States, such as txt4baby, are largely missing from the evidence base and little is known about participant experiences with these types of interventions. To better understand participant experiences within a large-scale, public health-focused, text message program targeting chronic illness, we conducted a multimodal survey of participants who had engaged in txt4health.

The purpose of this 2-part investigation is to evaluate the txt4health pilot studies in Southeast Michigan and Greater Cincinnati through the lens of the RE-AIM framework. In the present paper (Part II), we seek to document program efficacy in terms of participant perceptions of program satisfaction and usage, as well as its effects on behavior change. In our companion paper (Part I), we sought to document program reach and adoption. In comparison to the majority of previous work that has focused on small-scale implementations of mHealth programs [4-6], this txt4health evaluation represents an effort to understand user perceptions of a program that is operating at scale.

##  Methods

### Overview

This evaluation of txt4health was conducted in 2 parts. In Part I, we conducted a retrospective records analysis of individual-level txt4health system usage data from participants in Southeast Michigan and Greater Cincinnati to determine intervention reach and participant adoption of the program. Findings from Part I of the evaluation are presented in the companion paper. In Part II, we conducted a multimodal user survey with Southeast Michigan Beacon Community (SEMBC) and Greater Cincinnati Beacon Collaborative (GCBC) txt4health users recruited through the program. This survey sought to understand participant perceptions of program satisfaction and use, and self-reported perceptions of behavior change as a result of using txt4health. Txt4health is an automated, personalized, interactive, 14-week text message service targeting vulnerable at-risk populations, designed to help people understand their risk for type 2 diabetes and become more informed about the steps they can take to reduce that risk. Txt4health has been more extensively described in the Part I companion paper, as well as in recent work by Abebe et al [9].

###  Program Evaluation Framework: RE-AIM

We used the RE-AIM framework to guide this evaluation of the txt4health pilots in Southeast Michigan and Greater Cincinnati. As previously described in the companion paper, RE-AIM incorporates the dimensions of reach, efficacy, adoption, implementation, and maintenance, and is a framework that has been extensively used to guide the planning, evaluation, reporting, and review of health promotion interventions [10]. Within the present paper, we focus strictly on the dimension of efficacy of the RE-AIM framework. Efficacy is an individual-level measure that refers to the degree to which use of an intervention creates the desired outcomes [10]. Within the context of the present txt4health evaluation, efficacy refers to participant perceptions of txt4health, as well as perceptions of the effect of txt4health on behavior.

###  Participant Recruitment

To be eligible to participate in this study, individuals had to be at least 18 years and enrolled in the txt4health program for at least 10 weeks. Beacon employees enrolled in txt4health were excluded from participation in this survey. For recruitment purposes, we sent a text message to all txt4health users in the SEMBC and GCBC pilots who were subscribed to the program at the time of solicitation. In total, 814 participants were active in the txt4health system when survey recruitment text messages were sent. Six batches of recruitment texts were sent to all active participants between September and December, 2012 (approximately every 2 weeks), and asked txt4health users to indicate whether or not they would be interested in participating in the survey. Those individuals who responded yes were contacted via phone to coordinate survey completion. Phone calls were not made to potential participants until at least 10 weeks had passed after a participants’ initial txt4health enrollment date. This allowed for participants to complete most of the program before taking the survey. Those individuals who responded no were removed from our solicitation list and they never received another recruitment text message. Participants who did not respond to the recruitment text messages remained on our solicitation list and continued to receive recruitment text messages. Up to 5 attempts were made by phone to contact all participants who expressed interest in survey participation.

Once contact was established, potential participants were again asked if they were interested in participating. Any individual no longer interested was thanked for their time. All eligible participants willing to participate were given the option of completing the survey via phone, Internet (survey hosted on SurveyMonkey [11]), or US Mail. Individuals who wished to take the survey via Internet or US Mail received periodic reminders via email or phone for up to 6 weeks, or until survey completion. All survey respondents were eligible to receive a US $10 gift card to a local retailer for completing the survey. The Wayne State University Institutional Review Board approved the user survey portion of this study with a waiver of documentation of written consent. In lieu of written consent, all participants who completed the survey were given information pertaining to the study, including study procedures, risks/benefits, and incentives. This information was distributed in electronic form for online survey participants, in hard copy for US Mail participants, and over the phone with a statement of oral consent for phone survey participants.

###  Measures

The investigator-developed user survey took approximately 20 minutes to complete and included questions assessing txt4health use and perceptions, health and health behaviors, mobile phone use, demographics, and activation as measured by the Patient Activation Measure (PAM). The PAM is a 13-item instrument that has been validated to assess individuals’ knowledge, skill, and confidence with managing their health. The PAM characterizes people into 1 of 4 levels of activation, with descriptions of each level as follows in Table 1 [12,13].

**Table 1:** Description of Patient Activation Measure (PAM) level categorizations.

PAM level	Description^a^
1	Does not feel in charge of their own health and care. Managing health is overwhelming for them with all of life’s other challenges. Lacks confidence in their ability to manage health. Has few problem-solving skills and poor coping skills. They may not be very aware of own behaviors.
2	May lack basic knowledge about their condition, treatment options, and/or self-care. Have little experience or success with behavior change. Look to their doctor to be the one in charge. Low confidence in their ability to manage health.
3	Have the basic facts of their conditions and treatments. Some experience and success in making behavioral changes. Some confidence in handling limited aspects of their health.
4	Have made most of the necessary behavior changes, but may have difficulty maintaining behaviors over time or during times of stress.

^a^Descriptions from PAM licensing materials packet from Insignia Health [13].

###  Data Analysis

We conducted descriptive statistics to describe participant characteristics, perceptions of txt4health, self-reported text4health usage, and self-reported effects of txt4health. Continuous variables were expressed as mean (SD) and means were compared using 2-tailed unpaired independent samples *t* tests. Categorical data were displayed as frequencies and percentages, and chi-square tests were used for comparison. It should be noted that because participants were not required to answer all items within the survey, the denominator of many of our analyses fluctuates slightly. Multiple regression analyses were used to predict satisfaction with txt4health controlling for the continuous variables age and body mass index (BMI), as well as the categorical variables gender, White or non-White race, income, education, level of activation as measured by the PAM, and Beacon Community affiliation (Southeast Michigan or Greater Cincinnati). Moreover, chi-square analyses were used to explore potential differences between participants affiliated with the Southeast Michigan or Greater Cincinnati Beacon Communities. Significance levels were set at a *P* value <.05. All statistical analyses were carried out using STATA version 11.0 (StataCorp LP, College Station, TX, USA).

## Results

### Overview

**Figure 1:**
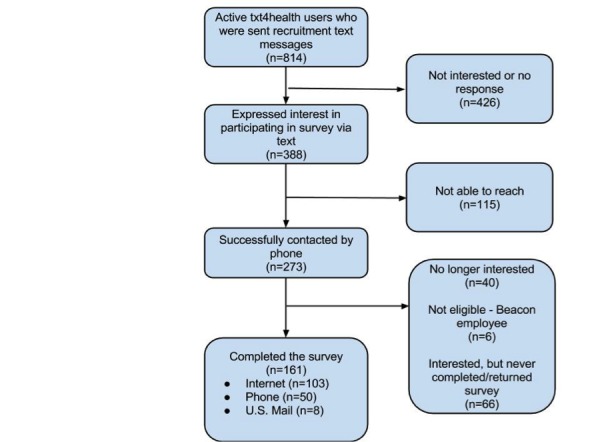
Participant flow.

We conducted a survey of Southeast Michigan and Greater Cincinnati txt4health participants recruited via text message. Of the 814 active txt4health participants who were sent recruitment text messages, 47.7% (388/814) expressed initial interest in participating in the survey via text message. We successfully contacted 70.4% (273/388) of those individuals by phone and asked again if they would be willing to participate. Overall, 14.7% (40/273) of txt4health users who previously expressed interest in our survey declined participation, and an additional 6 (2.2%) individuals were excluded from participation because they were Beacon employees. Of the remaining 227 eligible individuals still interested in survey participation, 70.9% (161/227) of participants completed the approximately 20-minute survey; 64.0% (103/161) completed it via the Internet, 31.1% (50/161) completed it via phone, and 5.0% (8/161) completed it via US Mail. Refer to Figure 1 for a complete chart of participant flow.

Of the 161 participants who completed the user survey, the sample was predominantly female (73.7%, 115/156), African American (51.6%, 81/157) or White (44.6%, 70/157), smartphone users (78.5%, 124/158), with a mean age of 42.4 years (SD 11.6) and a mean BMI of 33.1 kg/m^2^ (SD 8.6). Most survey respondents were obese with a BMI ≥30 kg/m^2^ (53.9%, 82/152), or overweight with a BMI between 25.0 and 29.9 kg/m^2^ (31.6%, 48/152). In addition, 30.6% (48/157) had a history of diabetes. Most survey participants (75.6%, 121/160) self-reported good, very good, or excellent health. Overall, this sample was well educated with 58.6% (92/157) reporting that they were college graduates or had completed postgraduate work, and an additional 28.0% (44/157) reporting that they had completed some college classes. Only 8.9% (14/157) reported that they were high school dropouts, or had completed high school. Annual income of survey participants was well distributed, with 22.2% (34/153) reporting annual household incomes of less than US $25,000, and 20.9% (32/153) with more than US $100,000. Regarding levels of activation, 63.5% (101/159) of respondents were categorized as level 4 on the PAM, which is characterized as individuals who “have made most of the necessary behavior changes, but may have difficulty maintaining behaviors over time or during times of stress” [13]. An additional 22.6% (36/159) were categorized as level 3 on the PAM, which is characterized as individuals who “have the basic facts of their conditions and treatments, some experience and success in making behavioral changes, and some confidence in handling limited aspects of their health” [13]. For 53.8% (85/158) of respondents, txt4health was the first text message program that they had used. See Table 2 for a complete breakdown of survey respondent characteristics.

**Table 2:** Characteristics of txt4health user survey respondents (N=161).

Participant characteristic		Southeast Michigan	Greater Cincinnati	Total
**Gender, n**		94	62	156
	Female, n (%)	65 (69.1)	50 (80.6)	115 (73.7)
	Male, n (%)	29 (30.9)	12 (19.4)	41 (26.3)
**Age (years), n**		93	62	155
	Mean (SD)	43.3 (11.8)	41.0 (11.3)	42.4 (11.6)
**Hispanic or Latino origin, n**		92	61	153
	Yes, n (%)	2 (2.2)	0 (0.0)	2 (1.3)
	No, n (%)	89 (96.7)	61 (100.0)	150 (98.0)
	Don’t know, n (%)	1 (1.1)	0 (0.0)	1 (0.7)
**Race,** ^a^ **n**		94	63	157
	White, n (%)	30 (31.9)	40 (63.5)	70 (44.6)
	Black or African American, n (%)	62 (66.0)	19 (30.2)	81 (51.6)
	Other, n (%)	2 (2.1)	3 (4.8)	5 (3.2)
	Don’t know, n (%)	0 (0.0)	1 (1.6)	1 (0.6)
**Income (US $), n**		92	61	153
	<25,000, n (%)	18 (19.6)	16 (26.2)	34 (22.2)
	25,000-49,999, n (%)	20 (21.7)	10 (16.4)	30 (19.6)
	50,000-74,999, n (%)	21 (22.8)	9 (14.8)	30 (19.6)
	75,000-99,999, n (%)	13 (14.1)	9 (14.8)	22 (14.4)
	100,000-124,999, n (%)	5 (5.4)	3 (4.9)	8 (5.2)
	≥125, 000, n (%)	10 (10.9)	14 (23.0)	24 (15.7)
	Don’t know, n (%)	5 (5.4)	0 (0.0)	5 (3.3)
**Education, n**		94	63	157
	Some high school, n (%)	2 (2.1)	0 (0.0)	2 (1.3)
	High school diploma or GED, n (%)	5 (5.3)	7 (11.1)	12 (7.6)
	Trade or vocational school, n (%)	5 (5.3)	2 (3.2)	7 (4.5)
	Some college, n (%)	27 (28.7)	17 (27.0)	44 (28.0)
	College graduate, n (%)	27 (28.7)	19 (30.2)	46 (29.3)
	Postgraduate work or degree, n (%)	28 (29.8)	18 (28.6)	46 (29.3)
**Where you go to for health care services, n**		93	62	155
	Family doctor/nurse or clinic, n (%)	82 (88.2)	53 (85.5)	135 (87.1)
	Emergency department, n (%)	4 (4.3)	4 (6.5)	8 (5.2)
	Urgent care clinic, n (%)	4 (4.3)	2 (3.2)	6 (3.9)
	Internet, n (%)	2 (2.2)	1 (1.6)	3 (1.9)
	Nowhere, n (%)	1 (1.1)	2 (3.2)	3 (1.9)
**Diabetes history,** ^a^ **n**		94	63	157
	Yes, n (%)	39 (41.5)	9 (14.3)	48 (30.6)
	Yes, but only during pregnancy, n (%)	2 (2.1)	3 (4.8)	5 (3.2)
	No, n (%)	44 (46.8)	43 (68.3)	87 (55.4)
	No, but prediabetes or borderline diabetes, n (%)	9 (9.6)	8 (12.7)	17 (10.8)
**Prediabetes history,** ^b^ **n**		84	56	140
	Yes, n (%)	20 (23.8)	4 (7.1)	24 (17.1)
	Yes, but only during pregnancy, n (%)	3 (3.6)	3 (5.4)	6 (4.3)
	No, n (%)	61 (72.6)	49 (87.5)	110 (78.6)
**BMI category, n**		91	61	152
	Normal, n (%)	11 (12.1)	11 (18.0)	22 (14.5)
	Overweight, n (%)	26 (28.6)	22 (36.1)	48 (31.6)
	Obese, n (%)	54 (59.3)	28 (45.9)	82 (53.9)

^a^Significant difference (*P*<.01) found between Southeast Michigan Beacon Community (SMBC) and Greater Cincinnati Beacon Collaborative (GCBC).

^b^Significant difference (*P*<.05) found between SEMBC and GCBC.

###  Survey Participants’ txt4health Use

Survey participants first learned of txt4health through a variety of channels, including health fairs (20.0%, 32/160), radio advertisements (14.4%, 23/160), and directly from health care providers (13.8%, 22/160). Despite the number of respondents who first learned of txt4health through health fairs where individuals were able to initiate enrollment by providing a phone number and ZIP code to Beacon staff members, only 4.4% (7/160) of respondents initiated enrollment through that mechanism. Rather, 92.5% (148/160) self-reported initiating enrollment through texting the word “health” to the txt4health short code. Regarding use of the program, 83.2% (134/161) of survey participants reported using txt4health to set physical activity goals, and 67.5% (108/160) reported setting weight loss goals. The vast majority of survey participants (82.5%, 132/160) reported that they always read the text messages they received from txt4health, and 26.3% (42/160) of survey participants reported discussing txt4health messages with their health care provider. One component of txt4health was the inclusion of messages regarding resources of potential interest, such as contact information for community resources and organizations, links to additional health-related information, etc. Survey respondents reported using these resources never (25.0%, 40/160), rarely (30.0%, 48/160), occasionally (26.3%, 42/160), or frequently/very frequently (15.0%, 24/160).

###  Survey Participants’ Perceptions of txt4health

All survey participants agreed/strongly agreed that the messages included in txt4health were clear and easy to understand (100.0%, 160/160). Most reported txt4health made them knowledgeable about their risk for type 2 diabetes (88.1%, 140/159) and the program made them conscious of their diet and physical activity habits (88.8%, 142/160). Most survey participants reported that they agreed/strongly agreed that they enjoyed participating in the program (88.1%, 140/159). Although 6.9% (11/159) indicated that there were too many messages sent in the program, most participants indicated that the number of messages sent each week was just the right amount (79.9%, 127/159). See Table 3 for a complete breakdown of participant responses to the txt4health perception items. Perceptions of satisfaction among survey participants were very positive with 67.1% (108/161) reporting satisfaction scores of 8 or higher on a 10-point scale, with 10 equivalent to most satisfied (mean 8.2, SD 1.6). Using multiple regression analyses, race was a significant predictor of satisfaction scores (beta=.81, *P*=.008) with non-White participants reporting higher mean satisfaction scores than White participants (mean 8.6, SD 1.5 and mean 7.7, SD 1.6, respectively; *t*_155_=–3.79, *P*<.001). In addition, PAM level was also predictive of satisfaction scores (beta=.33, *P*=.03) with individuals at PAM level 4 rating their satisfaction higher than those with PAM levels less than 4 (mean 8.5, SD 1.6 and mean 7.7, SD 1.6, respectively; *t*_157_=–2.96, *P*=.004).

**Table 3:** Participant response to behavior change items.

Item		Disagree/strongly disagree n (%)	Neutral n (%)	Agree/strongly agree n (%)
**Perceptions of txt4health**				
	The text messages were clear and easy to understand (n=160)	0 (0.0)	0 (0.0)	160 (100.0)
	Txt4health made me knowledgeable about my risk for type 2 diabetes (n=159)	4 (2.5)	15 (9.4)	140 (88.1)
	Txt4health made me conscious of my diet and physical activity habits (n=158)	4 (2.5)	12 (7.6)	142 (89.9)
	Txt4health helped me become more physically active (n=158)^a^	17 (10.8)	35 (22.2)	106 (67.1)
	Txt4health helped me lose weight (n=160)	37 (23.1)	50 (31.3)	73 (45.6)
	I enjoyed participating in the program (n=156)^a^	1 (0.6)	15 (9.6)	140 (89.7)
	Text4health helped improve the way I manage my mental health (n=157)	26 (16.6)	50 (31.8)	81 (51.6)
**Behavior change**				
	After having completed txt4health, I am more likely to replace sugary drinks such as juice or soda with water (n=158)^a^	8 (5.1)	26 (16.5)	124 (78.5)
	After having completed txt4health, I am more likely to have a piece of fresh fruit instead of dessert (n=158)	7 (4.4)	33 (20.9)	118 (74.7)
	After having completed txt4health, I am more likely to substitute a small salad for chips or fries when dining out (n=159)	10 (6.3)	28 (17.6)	121 (76.1)
	After having completed txt4health, I buy healthier foods when grocery shopping (n=158)^a^	4 (2.5)	28 (17.7)	126 (79.7)
	After having completed txt4health, I eat more grilled, baked, or broiled foods instead of fried (n=158)	5 (3.2)	33 (20.9)	120 (75.9)

^a^Does not equal 100% due to rounding error.

###  Self-Reported Behavior Change Attributed to txt4health

Because the goal of txt4health is to promote behavior change among participants, survey respondents were asked to reflect on how txt4health may have changed their behavior. The majority of the participants agreed/strongly agreed that after having completed txt4health, they were more likely to replace sugary drinks, such as juice or soda, with water (78.0%, 124/159), have a piece of fresh fruit instead of dessert (74.2%, 118/159), substitute a small salad for chips or fries when dining out (76.1%, 121/159), buy healthier foods when grocery shopping (79.7%, 126/158), and eat more grilled, baked, or broiled foods instead of fried (75.5%, 120/159). Of those individuals who set goals within txt4health, most reported meeting their physical activity goals most of the time or always (60.6%, 80/132), whereas 25.7% (28/109) reported meeting weight goals most of the time or always. In addition, most (66.3%, 106/160) agreed/strongly agreed that the program helped them become more physically active, and almost half reported that txt4health helped them to lose weight (45.6%, 73/160). See Table 3 for a complete breakdown of participant response to behavior change items.

##  Discussion

Among txt4health users who participated in the user satisfaction and perceptions survey, participants reported very positive perceptions of txt4health. Overall, the majority of survey participants reported enjoying the program and rated their satisfaction as 8 or higher on a 10-point scale, with 10 equivalent to most satisfied. In addition to positive perceptions of the program, survey respondents self-reported making behavior choices related to diet and physical activity as a result of their participation, and approximately half of respondents self-reported that txt4health helped them to lose weight. Many of these positive perceptions were echoed in the open-ended response section of the survey, in which participants were given an opportunity to provide additional comments. Overall, the tone of these comments was positive, and survey respondents offered helpful and constructive suggestions on how to improve the program rather than any overtly negative feedback. Given our recruitment strategy solicited individuals who were active within the program and excluded any individuals who dropped out before recruitment, it is not surprising that participant response regarding the program was overwhelmingly positive. Undoubtedly, this recruitment strategy introduced a fair amount of bias into the survey results because only individuals who had not dropped out of the program received the invitation to participate. This, coupled with the self-selection bias that occurs from participants who were likely skewed toward favoring txt4health, is potentially problematic. Future research should seek to conduct an evaluation with a stronger research design that includes random sampling of both active and dropout participants to reduce the amount of bias within the sample.

Despite these limitations related to sampling, it is clear that for a subset of individuals who initially enrolled in txt4health, this approach to a behavior change program was met with great satisfaction from many participants, and suggests that this is a feasible and acceptable method to promote behavior change to large numbers of individuals who may derive some perceived benefit from participation. There appears to be a relatively discrete subset of individuals who responded very well to this type of program in terms of both program usage (see our companion paper, Part I) and overall perceptions of txt4health. In particular, it appears that minority participants respond more favorably to the txt4health intervention. Results from Part I of this investigation showed that non-White participants stayed in txt4health longer than White participants (see Part I companion paper), and results from the present user survey show that non-White participants had higher ratings of satisfaction than White participants.

Because it is unlikely that there exists a one-size-fits-all approach to behavior change programs that will be met with universal acceptance, future work should seek to understand which types of people are best suited to different types of programs, as well as to develop instruments for identifying these individuals. This may allow for better targeting of programs to those individuals who are likely to derive the most benefit. Throughout our analysis, demographic characteristics seldom predicted participant perceptions or usage of txt4health. This suggests that with the exception of non-White race predicting length of participation and satisfaction, demographics are not the drivers of use/acceptability in text message–based programs, and perhaps more psychological factors may be key.

In addition to positive perceptions of txt4health among survey respondents, many participants also self-reported that participation in the program led to positive behavior changes, which we hope will ultimately lead to positive health outcomes. Recent work by Ramachandran et al [14] supports the use of text messaging to gain positive health outcomes related to diabetes. In that study, 537 working men in India were randomized to a text message program (similar to txt4health) or usual care. The authors found after 2 years of program use, the text message program was effective at preventing type 2 diabetes; 27% of control group participants developed type 2 diabetes by the end of the trial compared to 18% of the text message group participants [14]. This recent finding in India has increased the desire to more concretely establish the efficacy of this type of program among Americans, and to see if these findings could be replicated here. Unfortunately, in the present study, because of the limitations of our research design, we lack objective measures that would reveal whether txt4health actually caused people to change their behavior; thus, we are not able to make a judgment regarding the efficacy of text4health on health outcomes. Rather, our evaluation relies on self-reported survey data that only provides limited insight into participant perceptions of the program. Reasons for the limitations regarding research design have been previously described [9], but briefly include issues related to program dissemination strategies, as well as limited time and financial resources. Although small-scale pilot studies exist that indicate text message programs can be effective at promoting behavior change, well-powered randomized controlled trials establishing efficacy of these types of programs among American adults are largely missing from the literature [4-6]. Given the participant response regarding self-reported behavior change from this study, future work should seek to test the efficacy of txt4health and similar broad-based, large-scale, public health text message interventions using well-thought-out experimental designs with appropriate controls.

Despite the proliferation of small-scale pilot studies demonstrating the efficacy of text message mHealth programs for chronic disease, little is known about user perceptions of these types of programs among American adults while operating at scale. Results from this study suggest that participants in txt4health, a large-scale public health–focused text message program targeting type 2 diabetes, have positive perceptions of the program and that participation has led to positive behavior change. Although these results come from a seemingly biased sample of txt4health users who self-selected to participate in the user survey, these findings do suggest that a large subset of individuals who participate in a broad-based, public health–focused intervention may respond quite favorably.

## Abbreviations

BMI: body mass index
GCBC: Greater Cincinnati Beacon Collaborative
mHealth: mobile health
PAM: Patient Activation Measure
SEMBC: Southeast Michigan Beacon Community
